# The Motor Subsystem as a Predictor of Success in Young Football Talents: A Person-Oriented Study

**DOI:** 10.1371/journal.pone.0161049

**Published:** 2016-08-10

**Authors:** Marc Zibung, Claudia Zuber, Achim Conzelmann

**Affiliations:** Institute of Sport Science, University of Bern, Bern, Switzerland; University of Toronto, CANADA

## Abstract

Motor tests play a key role in talent selection in football. However, individual motor tests only focus on specific areas of a player’s complex performance. To evaluate his or her overall performance during a game, the current study takes a holistic perspective and uses a person-oriented approach. In this approach, several factors are viewed together as a system, whose state is analysed longitudinally. Based on this idea, six motor tests were aggregated to form the *Motor Function* subsystem. 104 young, top-level, male football talents were tested three times (2011, 2012, 2013; *M*_age_, t_2011_ = 12.26, *SD* = 0.29), and their overall level of performance was determined one year later (2014). The data were analysed using the LICUR method, a pattern-analytical procedure for person-oriented approaches. At all three measuring points, four patterns could be identified, which remained stable over time. One of the patterns found at the third measuring point identified more subsequently successful players than random selection would. This pattern is characterised by above-average, but not necessarily the best, performance on the tests. Developmental paths along structurally stable patterns that occur more often than predicted by chance indicate that the Motor Function subsystem is a viable means of forecasting in the age range of 12–15 years. Above-average, though not necessary outstanding, performance both on fitness and technical tests appears to be particularly promising. These findings underscore the view that a holistic perspective may be profitable in talent selection.

## Introduction

In order to select talents in football, it is essential to determine the players’ potential performance [1]. As it is difficult to assess their complex performance in the game objectively, one often resorts to fitness and technical tests in practice [2], since these are generally regarded to provide a high degree of objectivity and reliability [3, 4]. In terms of their validity, such tests ought to be able to distinguish between players displaying different levels of proficiency (concurrent validity), which they are generally considered to do [5]. However, when diagnosing talent, the difference in performance that will occur *in the future*—ideally when reaching the age of a competitive athlete—is of particularly great interest. The tests should therefore also be able to discriminate between players with different levels of potential (predictive validity). The question whether the fitness and technical tests currently used are able to meet these requirements has so far remained largely unanswered [2].

In football, top performance requires a combination of excellent fitness skills and the greatest possible technical precision. The fitness skills that are particularly important include speed [6] (starting speed, speed of reaction, speed persistence, agility) and (intermittent) endurance [7]. Among the technical skills, particularly dribbling, passing, and ball handling appear to contribute substantially to explaining performance [1]. Against this background, it immediately becomes clear that a talent diagnosis that is based on the results of individual motor test will be inadequate. If only individual test results and their connection to sports performance are investigated, then the interactions between different characteristics, and the possibility of their compensating for each other, will not be taken into account [8]. On top of this, it does not seem plausible that individual variables should be able to predict a complex developmental process. Corresponding evidence is also found in developmental psychological research (as already noted by Wohlwill, [9]).

In most cases, empirical studies and practical talent selection procedures have aggregated several variables or test outcomes in order to obtain as comprehensive a picture of the individual player as possible [10]. However, the question that arises here is *how* this aggregation should be done. This is the starting point for the current paper. So far, empirical studies aiming to assess the quality of the individual variables or test performance scores have developed models and tested theses using methods based on the *General Linear Model* (GLM) [11, 12]. Although GLM allows us to determine the variance attributable to individual variables, two problems still remain unresolved.

Firstly, such procedures generally assume a linear relationship between independent and dependent variable, an assumption that often turns out to be questionable [13]. When looking at the motor test results, for example, it is necessary to critically question whether the performance in endurance tests is linearly related to a player’s performance on the pitch. Reilly et al. [10] have been able to demonstrate relatively large differences in the endurance of top football players. From a methodological point of view, other relationships could also be modelled, for example curvilinear models. However, in most cases, the nature of the relationship is not adequately explained on a theoretical level, and it may often even be doubtful whether the requirement of continuity is fulfilled. In fact, one should not rule out *a priori* the possibility of having step functions and thus discontinuous relationships.

Secondly, a further assumption frequently made is that the same relationships apply to all individuals. However, it seems more plausible that the relationship between a certain characteristic and the criterion (of potential performance as a player) can only be assessed once the intra-individual degrees of expression of the other characteristics have been taken into account. To do justice to such an intra-individual perspective, it is necessary to resort to alternative concepts and methods.

Since the fundamental questions of talent research are always related to processes in human development, it seems reasonable to base them on developmental scientific concepts. Modern developmental science takes a dynamic interactionist perspective (for an overview, cf. [14; 15]; in sports science: [16]). Magnusson and Cairns [17] in addition assume a holistic perspective of human development, which leads to a person-oriented approach [18]. This holistic approach is able to describe the development of an individual comprehensively, i.e. in the case of talent promotion, the entire process of developing from a promising young talent to a top athlete. The developing individual and his or her environment are viewed as a system, which can be divided up into various operationalisable subsystems. The interacting variables of a (sub-)system are called operating factors [19]. As the person-oriented approach does not assume linear relationships, it has to dispense with methods based on the GLM. For an overview and comparison of the variable- vs. person-oriented approach, cf. Bergman and Andersson [20].

More recently, talent research has favoured multidimensional approaches [1, 21, 22] which adopt a perspective that focuses on the development process of the talent [23, 24]. The person-oriented approach considers both, a multidimensional as well as a developmental perspective in theoretical and methodological respects. So far, it has been successfully applied in sports talent research within the subsystems *Training* [25] and *Motivation* [26], with promising results. The idea therefore arises naturally to apply the person-oriented approach to fitness and technical skills, too, to develop a better understanding of the intra-individual relationship between different test performance scores and ultimately success.

## The Present Research

The present longitudinal study uses a person-oriented approach to map patterns of fitness and technical test performance scores of young talented football players and identifies developmental types that are particularly promising in the medium term in adolescence. Furthermore, it examines the stability of these development patterns, since it is of key importance to talent selection and promotion. If the same or similar patterns keep emerging over time (structural stability), it follows that the same types can be distinguished at different times when identifying talents. If certain developmental pathways are particularly frequent on the individual level (individual stability) and also associated with success in sports, promoting players of this type will be particularly promising. If individual stability follows structurally stable patterns, then it is fair to assume, in addition, that the time at which the type is determined does not matter, which would be particularly valuable to talent selection.

The analysis is guided by the following questions:

1. Which patterns can be identified in young football talents in terms of their fitness skills and their technical skills?
2. Can the same or similar patterns be recognised again after one or two years (structural stability)?
3. Which developmental paths do the top talented football players follow during this time (individual stability)?
4. Do patterns exist that coincide particularly with sports success a year later?

## Method

### Participants and procedure

The sample consists of *N* = 136 top talented male football players who were members of various regional teams of the Swiss Football Association at the first measuring point (2011) (*M*_*age*, *t1*_ = 12.26, *SD* = 0.29). The players took part in three testing events at one-year intervals, at which motor tests were conducted. The data analysis only took into account players who were present for at least two of the three testing sessions. In addition, outliers have been removed (see chapter Residual Analysis) so that the final sample includes *n* = 104 players. A year after t_3_, the talent cards assigned by the Swiss Football Association were used as an indicator of the level of performance reached by the individual players. The topmost talented players are assigned national talent cards, while other talented players receive regional talent cards. This allows three levels of performance to be distinguished: players with (1) a national, (2) a regional, or (3) no talent card.

The participants and their parents provided their written informed consent to participate in this study. Before each data collection session, all the participants were asked whether they wanted to participate and were informed that they could discontinue at any time during the study. All data were treated confidentially. The study including the informed consent forms was approved by the ethics committee of the Philosophical Humanist Faculty at the University of Bern.

### Measures

In addition to the three fitness and three technical tests described below, age, height, and weight were ascertained as additional variables, as well as biological maturity on the basis of the Mirwald Test [27].

#### Yo-yo test

The intermittent endurance performance was measured by means of the yo-yo intermittent recovery test [11]. In this test, players run back and forth between two lines, 20 metres apart, at a pace dictated by acoustic signals. Between two laps, there is a pause of 10 seconds. The speed is gradually increased. The test score is the total distance covered up until the last level reached before dropping out.

#### Sprint test

Sprinting speed was measured using a 40-metre sprint test without a starting signal. A twin photoelectric sensor at the starting line triggers the stopwatch and an identical photoelectric sensor stops the clock at the 40-metre line.

#### Agility test

In the agility test, the players take a short sprint (without a start signal), then run around three poles (right, left, right), then do an intermediate sprint, and finally negotiate three further poles in reverse order (left, right, left) before sprinting over the finishing line [28]. Times are measured using photoelectric sensors, as during the sprint test.

#### Dribbling test

The dribbling test [28] is set up and carried out identically to the agility test, the only difference being that it is performed with rather than without a ball.

#### Passing test

In the passing test (adapted from [28]), players pass the ball as quickly as possible from a confined zone to bounce it off four walls in turn, one in each direction. After the fourth pass, the same sequence is repeated in reverse order (reaching a total of nine passes). The time was measured manually with stopwatches.

#### Juggling test

In the juggling test, [28] players take turns juggling with their left and right foot along a marked course shaped like the figure 8. For each quarter of a circle they complete, they are given a point. The test is stopped as soon as a mistake is made (e.g. left foot twice in succession, or ball touching the ground) or at the latest after 45 seconds.

With the exception of the yo-yo test, players were given two attempts at each test, whereby the better of the two was used for the analysis. All the tests were carried out by a trained team of testers, exclusively on synthetic turf, following a standardised procedure. In the event of rain, the tests were postponed or interrupted and carried out at a later time.

### Data analysis

Since complete sets of data were required for the subsequent cluster analyses, the missing values were imputed. Little’s MCAR (missing completely at random) test led to a non-significant result (*χ*^2^ = 230.3, *df* = 197, *p* = .052), meaning that the missing data are completely at random and imputation using Expectation Maximization (EM) is therefore permissible [29, 30]. Overall, 22.5% of the data were imputed by EM.

#### LICUR method

The fundamental consequence of abandoning the *GLM* has already been discussed in connection with the methodological implementation of the person-oriented approach. The LICUR method (**Li**nking of **C**l**u**sters after removal of a **R**esidue, cf. [19]) is a pattern-analytical procedure that is suitable for implementing person-oriented approaches. The fundamental idea behind it is to form groups (clusters) within individual phases of development, which contain subjects displaying similar levels of the operating factors (patterns). Afterwards, the individual transitions from one cluster to the next in the following phase or to a certain group with a specific developmental outcome is determined. By comparing the number of transitions expected with the number actually observed, transitions that occur more frequently than by chance alone (so-called developmental types) and less frequently than expected (so-called developmental antitypes) can be identified [31]. The LICUR method is employed in three steps [19]: first, a residual analysis is conducted. In this, extreme cases (residues) are identified and removed from the data set as they would otherwise distort the cluster solution. Next, clusters are formed (cluster analysis) for each measuring point (t_1_, t_2_, t_3_), and subjects are assigned to these. Finally, similarities in the patterns between different phases are investigated, with special attention to the frequencies with which subjects make the transitions between them, which eventually determines the developmental (anti-)types. The first and third steps were followed using the statistics package SLEIPNER 2.1 [32], whereas the cluster analysis was performed using IBM SPSS Statistics 22.0.

#### Residual analysis

During residual analysis, the patterns of pairs of subjects are compared. Any subject not displaying similarities with at least a predetermined number of other subjects (squared Euclidian distance) is identified as being a residue. According to Bergman et al. [19], the number of residues should not exceed 3% of the total sample size. For the analysis, a threshold value of *T* = 1.2 for the distance was chosen. The minimum number of similar cases was set to be *K* = 1, meaning that only those subjects were excluded whose pattern is unique.

#### Cluster analysis

The Ward procedure using the squared Euclidian distance as a measure of separation was chosen for the cluster analysis, as recommended in the literature on person-oriented approaches [19, 33] and as also considered the standard for most other applications of cluster analyses [34]. The optimum cluster solution was determined based on contents (interpretability of the clusters) as well as statistical criteria. The statistical criteria included the elbow criterion as well as the Mojena test [34] with a threshold value of 2.75. Furthermore, solutions with the smallest number of an *F* scores >1 were favoured [35]. Afterwards, the cluster solutions that had been found were then optimised by subjecting them to a cluster centre analysis [35].

#### Structural stability

Structural stability (*SS*) refers to the similarity of the patterns found at different measuring points. If the patterns are replicated in similar forms (i.e. similar cluster centroids are found), they are said to display high structural stability. To analyse the structural stability, the average square Euclidian distance between clusters is compared. The clusters are arranged in pairs with increasing scores, which means that the most similar clusters are located at the same level alongside each other.

#### Individual stability (developmental types)

To analyse the individual development paths, the transitions from the clusters of one phase to the clusters of the next phase or to a specific development outcome are counted and checked for significant deviations from the assumption of randomness (*p* <.05) using the exact Fisher 4-field distribution test, based on a hypergeometric distribution [19]. Paths that occur more often than suggested by chance are defined as developmental types, and less common paths as developmental antitypes. The odds ratio indicates how much the probability of a particular developmental path is increased (developmental types) or decreased (developmental antitypes).

## Results

**Fig 1:**
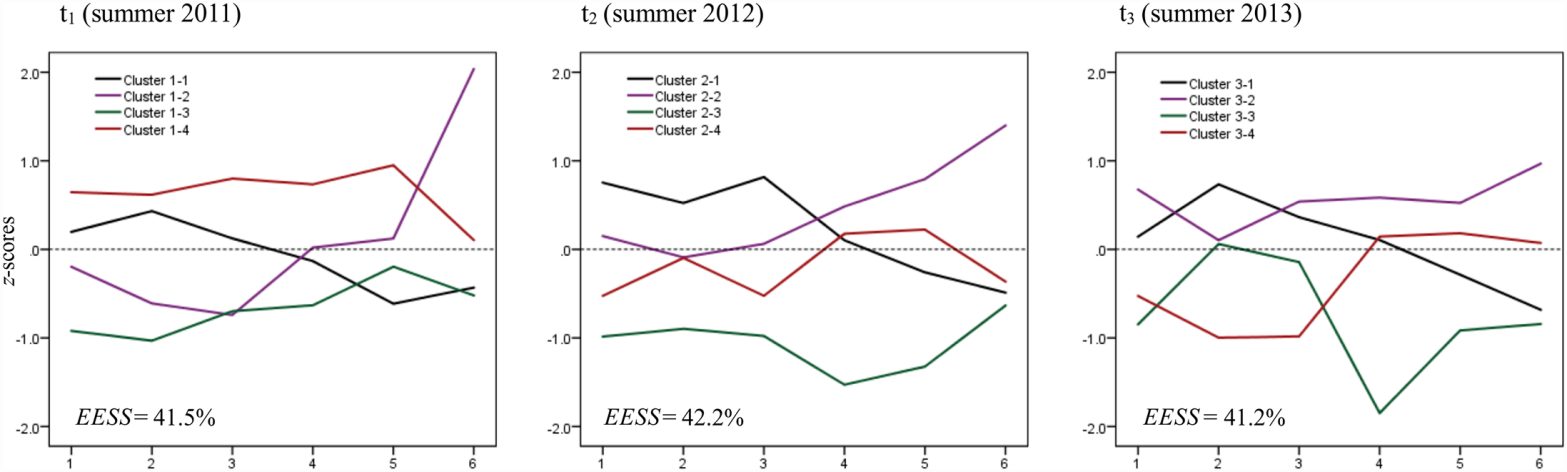
*z*-standardised cluster centroids at all three measuring points. Operating factors on the x-axis: 1 = Yo-yo test, 2 = Sprint test, 3 = Agility test, 4 = Dribbling test, 5 = Passing test, 6 = Juggling test. The test results have been adjusted such that for all variables a positive value indicates above-average performance. *EESS* = Explained Error Sum of Squares.

Initially, two residues (#1, #53) were identified in phase one but none in phases two and three. This number lies below the 3% limit, and the residues are reasonable in terms of their content. In the subsequent cluster analysis, the stated criteria indicated that a 4-cluster solution was the optimum at all three measuring points. After the cluster centre analysis, the final cluster solutions display an explained error sum of squares (*EESS*) of 41.5% at the first, 42.2% at the second, and 41.2% at the third measuring point (Fig 1).

Table 1 provides an overview of the descriptive statistics of the six operating factors for all the clusters at the three measuring points.

**Table 1:** Descriptive statistics (mean and standard deviation) of the operating factors.

	Operating factors												Additional variables (selection)^1^			
Measuring Point 1	Yo-yo test (metres)		Sprint test (seconds)		Agility test (seconds)		Dribbling test (seconds)		Passing test (seconds)		Juggling test (points)		Height (centimetres)		Biological maturity^2^	
	*M*	*SD*	*M*	*SD*	*M*	*SD*	*M*	*SD*	*M*	*SD*	*M*	*SD*	*M*	*SD*	*M*	*SD*
Total (*n* = 104)	811.8	263.2	6.62	0.34	8.19	0.35	10.67	0.61	18.70	2.07	2.58	2.96	151.8	7.3	2.74	0.61
Cluster 1–1 (*n* = 38)	863.9	199.0	6.47	0.25	8.15	0.26	10.75	0.52	19.97	1.72	1.30	1.49	152.7	8.3	2.74	0.60
Cluster 1–2 (*n* = 13)	760.0	287.1	6.82	0.21	8.45	0.23	10.66	0.60	18.44	1.60	8.62	2.66	150.2	4.5	3.00	0.00
Cluster 1–3 (*n* = 25)	569.6	167.4	6.97	0.27	8.43	0.31	11.05	0.61	19.10	1.91	1.04	.61	152.0	7.3	2.48	0.65
Cluster 1–4 (*n* = 28)	981.4	243.1	6.41	0.25	7.92	0.29	10.22	0.44	16.73	1.20	2.89	2.20	151.0	7.1	2.86	0.65
Measuring Point 2																
Total (*n* = 104)	1064.1	357.9	6.44	0.33	8.09	0.30	10.30	0.52	16.88	1.31	6.46	5.09	158.1	8.3	2.95	0.80
Cluster 2–1 (*n* = 34)	1333.8	349.9	6.27	0.29	7.85	0.21	10.24	0.39	17.22	1.04	3.97	2.73	157.4	9.1	3.06	0.88
Cluster 2–2 (*n* = 26)	1117.8	308.1	6.47	0.25	8.08	0.23	10.05	0.36	15.84	1.10	13.57	3.67	157.2	7.2	3.04	0.71
Cluster 2–3 (*n* = 14)	711.5	142.3	6.74	0.41	8.38	0.29	11.10	0.72	18.61	1.02	3.23	2.72	157.0	9.6	2.85	0.80
Cluster 2–4 (*n* = 30)	876.5	201.4	6.47	0.30	8.25	0.21	10.21	0.28	16.59	0.82	4.61	2.70	160.6	7.5	2.76	0.78
Measuring Point 3																
Total (*n* = 104)	1379.1	339.9	6.30	0.31	8.11	0.30	10.25	0.60	16.04	1.22	7.44	6.76	164.5	7.5	3.13	0.88
Cluster 3–1 (*n* = 31)	1427.9	225.9	6.07	0.24	8.01	0.26	10.19	0.36	16.39	1.01	2.83	2.11	164.6	8.7	3.00	0.69
Cluster 3–2 (*n* = 32)	1608.6	313.5	6.27	0.25	7.95	0.22	9.90	0.38	15.40	1.12	13.98	5.29	163.7	7.5	3.40	0.82
Cluster 3–3 (*n* = 14)	1091.3	251.4	6.28	0.24	8.16	0.29	11.36	0.49	17.16	1.17	1.74	3.77	166.7	7.1	3.25	0.71
Cluster 3–4 (*n* = 27)	1200.2	323.4	6.60	0.21	8.41	0.20	10.17	0.39	15.82	1.08	7.93	6.31	164.3	6.7	2.88	1.15

In the numbering of the clusters, the first digit stands for the measuring point, and the digit after the hyphen numbers the clusters within a phase from 1 to 4.

^1^ For the additional variables, missing values were not imputed, so that the number of cases per cluster are in some cases lower.

^2^ Mirwald test, scale of 1 to 5; 1 = early development, 5 = late development

The test results (Table 1) indicate that, as expected, the test results of the players improved from one measuring point to the next. Only the agility test did not display any improvement between t_2_ and t_3_. Fig 1 depicts the means as profiles of *z*-standardised values for each cluster. In the analysis of the profiles, it will be noted that there are large differences between the individual cluster profiles at a single measuring point. At t_1_, there is one cluster that has above-average scores across all operating factors (1–4), whereas another one has below-average scores (1–3) throughout. Cluster 1–1 has above-average scores on the fitness tests, but below-average scores on the technical tests. The profile of Cluster 1–2 is the exact opposite. Over the course of time (t_2_ and t_3_), the various clusters start resembling each other in terms of their profile, but their heights shift or change with regard to the extent of the operating factors.

**Fig 2:**
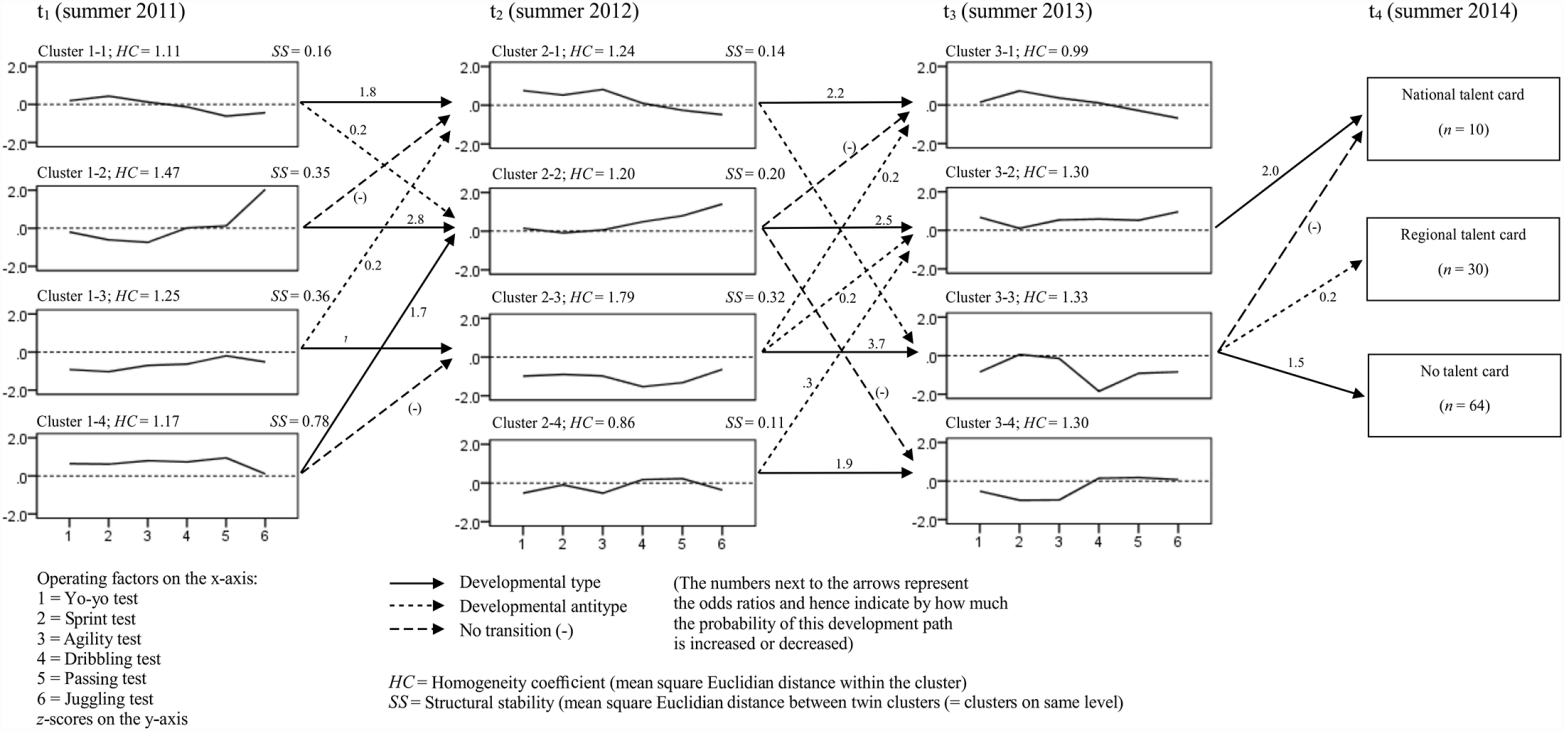
*z*-score profiles of the clusters (cluster centroids) and developmental (anti-)types for t_1_, t_2_, and t_3_ and the performance level at t_4_.

With regard to the first question, it will be noted that four patterns were identified at each of the three measuring points (Fig 1). The clusters are relatively homogeneous at all three measuring points, as demonstrated by the low values of the homogeneity coefficients (*HC*) (0.86 ≤ *HC* ≤ 1.79, Fig 2). Concerning the second question, the clusters are found to be replicated in a similar form at subsequent measuring points. This means they display a high level of structural stability (*SS*) (the measured distances lie between 0.11 ≤ *SS* ≤ 0.78). The four existing patterns of fitness and technical test performance scores change only slightly within two years. Concerning the additional characteristics measured (age, height, weight, and maturity), no differences can be found between the clusters.

To answer the third question under investigation, Fig 2 depicts all the developmental (anti-)types from t_1_ to t_3_. Three of the four developmental types between t_1_ and t_2_ follow structurally stable clusters. A further developmental type leads from Cluster 1–4 to 2–2, both of which clusters are also fairly similar in terms of their profiles. Developmental types between dissimilar clusters cannot be observed, which indicates that major changes in the profiles over the period of a year are fairly rare. The developmental antitypes between all the structurally dissimilar clusters support this finding. Paths along which no transitions took place are also marked, which is again observed to occur between dissimilar clusters, as might be expected. The same is observed with the developmental (anti-)types between t_2_ and t_3_; however, this behaviour is even more pronounced here: all four developmental types follow similar clusters. In other words, the individual stability follows structurally stable patterns. This means that the observed values for the motor subsystem remain largely steady over the period of time investigated, and that the players studied also remain in these groups.

Turning to the fourth question posed in this paper, which is particularly important in terms of talent development and talent selection, the transition probabilities from t_3_ to the three levels of performance are of special interest. One developmental type is found from Cluster 3–2, whose cluster centroid is the only one at this measuring point to display above-average values on all factors, to the group of the most successful players (national talent card). The odds ratio of 2.0 means that twice as many players from this cluster receive a national talent card as would be expected in a random distribution. A second developmental type leads from Cluster 3–3, characterised by almost entirely below-average scores on the operating factors, to the group of unsuccessful players (no talent card). Not a single player from this cluster holds a national talent card one year later. Fewer players from this cluster have a regional talent card after one year than chance would predict (developmental antitype).

## Discussion

In this study, young top promising football players were tested longitudinally at one-year intervals via three general fitness tests and three football-specific tests. The data were then analysed with a person-oriented approach using the LICUR method, a *pattern-analytical* procedure. This procedure focuses not on individual variables, but on several variables which together form a so-called (sub-)system, corresponding to the use of multidimensional approaches in talent research. We are interested in the constellation of the operating factors which form a pattern, rather than in the sums or means of the variables. In the present study, four structurally stable patterns were found for the Motor Function subsystem, indicating that the patterns re-emerge in a similar form at the next measuring point a year later. The observed developmental types (significantly higher transition probabilities) follow similar patterns, whereby a high correspondence between structural and individual stability is noted. This means that the relative proportions of the test performances remain stable over the two years under investigation. This correspondence is even clearer for the transition from t_2_ to t_3_. Since the variance in the maturity increases continuously from t_1_ to t_3_ (Table 1), this increasing stability cannot be explained in terms of a homogenisation of biological age over time. Instead, the causes lie in the structural circumstances. At the first measuring point, the players were selected for regional teams for the first time. The systematic process of talent promotion had only just begun. It can therefore be assumed that the performance-related heterogeneity was greater at this point in time than it was at a more advanced stage of talent promotion, after one or two years. Looking at a cross-section of the data, a decreasing variance is indeed observed over time for most of the test results (Table 1).

The starting point for this paper was that of using a holistic, person-oriented approach to identify patterns of fitness and technical test performances capable of predicting success. It follows that the key focus should lie on the final outcome: the transition probabilities from t_3_ to the subsequent levels of performance. The LICUR analysis shows that the pattern with above-average scores on all operating factors is the most promising in the short and medium term. However, if the strength of the operating factors is considered on its own, patterns are found which display higher scores on individual factors. For example, players from Cluster 3–1 are faster on average than the players in the most promising Cluster 3–2. This means that if a variable-centred perspective had been adopted and speed were viewed as a one-dimensional selection criterion, the choice made would be different. The pattern analysis reveals, however, that the players who perform above average in all the tests are later the most successful. Outstanding performance in individual areas along with below-average performance in other tests (Cluster 3–1) appears to be less promising—at least up to the age of 15. As might be expected, below-average to at best average performance in all tests (Cluster 3–3 and 3–4) is not that promising.

A developmental type can be identified across all three measuring points, following a structurally similar pattern displaying above-average scores on all operating factors (1–4, 2–2, 3–2). Since the clusters do not differ in terms of the additionally measured characteristics (especially in terms of biological maturity), this pattern is independent of the level of physical maturity. In addition, it is relatively stable on an individual scale since the number of transitions along these clusters exceeds those dictated by chance. The pattern therefore appears to be promising, independently of the time at which it is measured. Consequently, it has a certain prognostic validity, which is highly relevant to talent selection in practical sports situations. At the first measuring point, a second cluster is found (1–2), which displays a developmental type to the most promising pattern at t_2_, whereby its odds ratio is in fact higher than that of the developmental type from Cluster 1–4. These are players whose fitness performance is as yet below average, but whose football-related skills are already above average. For talent selection based on motor patterns, this suggests that the fitness factors at the first measuring point (age 12) are less relevant and therefore can be assigned less weight. Different scores in the fitness factors combined with above-average scores for skills specific to football are also more likely to lead to success in the long run.

In conclusion, the consensual demand for multidimensional and dynamic approaches to talent research have been implemented theoretically and methodologically. The findings show that studies based on the person-oriented approach also deliver useful results in the field of fitness skills and technical skills. At the same time, certain limitations in the study should also be noted. These primarily concern missing values and the imputation of data associated with these. Longitudinal studies in competitive sports are particularly affected by non-responses: injury, de-selection, trial training sessions, or medical appointments are just a few of the reasons for not taking part in the tests. Despite careful maintenance of the sample, 22.5% of the data had to be imputed. However, since the non-responses are random, imputation is permissible from a methodological point of view, and the results are in principle valid. Furthermore, it must be noted that the results are dependent on the sample in terms of the age of the players. Hence in the future it will be necessary to determine whether the patterns also remain stable during the subsequent career phase (starting at the age of 15–16 years) or whether perhaps other patterns emerge. This will require further longitudinal data covering even longer periods of time.

## Supporting Information

S1 File SPSS File containing row data. SPSS data file containing row data of all measuring points.
(SAV)
